# On research into the relationship between personality traits and the sporting level of competitive, professional and elite athletes

**DOI:** 10.3389/fpsyg.2024.1428107

**Published:** 2024-09-11

**Authors:** Paweł Adam Piepiora, Petra Čaplová, Paweł Zimoń, Róża Gumienna

**Affiliations:** ^1^Faculty of Physical Education and Sports, Wroclaw University of Health and Sport Sciences, Wrocław, Poland; ^2^Faculty of Science, Humanities and Pedagogy, Technical University of Liberec, Liberec, Czechia; ^3^Faculty of Medical and Technical Sciences, Karkonosze University of Applied Sciences, Jelenia Góra, Poland

**Keywords:** big five, NEO-FFI, neuroticism, sports psychology, sport theory

## Abstract

Data on the relationship between personality traits and athletes’ level of sportsmanship are not sufficiently documented. Therefore, it is reasonable to look for differences in personality traits between athletes from different levels: amateur, competitive and professional, as these groups of athletes function differently on a daily basis. Therefore, the aim of this article was to gain knowledge about the relationship between personality traits and the sporting level of athletes. The experiment examined male and female athletes (*N* = 119) aged 19–34, including 100 Polish professional athletes: 30 basketball players, 40 football players, 30 kyokushin style karate competitors; and 19 professional athletes (among them were the elite: 3 Olympic medallists): 4-person Polish Biathlon Team, 7-person Polish Luge Team, 8-person Swiss Mountain Bike (MTB) Team. The NEO-FFI Personality Questionnaire was used. Analyses were performed with the IBM SPSS Statistics 29.0 package at a statistical significance of α = 0.05. Significant differences were found in comparisons between groups of athletes: football players and karate competitors (in severity of neuroticism, extraversion, agreeableness, conscientiousness), football players and basketball players (in severity of neuroticism, extraversion, openness to experience, conscientiousness), football players and lugers (in severity of agreeableness), football players and mountain bikers (in severity of neuroticism). One significant difference was noted in the comparisons between athletes from different levels: competitive athletes had higher neuroticism severity than elite athletes. A weak and negative correlation between neuroticism and sporting levels was verified. But no correlation was shown between personality traits and the likelihood of becoming a professional. It was concluded that the observed differences between the studied groups of athletes could be derived from the specifics of the different sports. The elite are characterised by a lower intensity of neuroticism in relation to the competitive athletes, which can be seen in the relationship: the lower the neuroticism, the higher the sporting level.

## Introduction

This article falls within the field of athlete personality research in sport psychology. A review of previous research findings has shown that this is an ever-present research direction (Shuai et al., 2023). To date, no clarity has been achieved in personality theoretical constructs in sport psychology, as many of the studies to date undertake different personality theories and research tools. A consensus, though, is that individual personality traits have a decisive impact on sport performance (Lin et al., 2011). In addition, personality traits differentiate athletes’ sport engagement and mental preparation styles (Allen et al., 2011; Piepiora et al., 2023). And athletes’ well-being is related to their personality (Terracciano et al., 2013). Personality traits also correlate with long-term sporting success (Allen et al., 2013; Trninić et al., 2016). Meanwhile, neuroticism is an important link between emotions and physiology in relation to anxiety, arousal and confidence (Balyan et al., 2016). Different sports require a slightly different intensity of personality traits relating to the specifics of a given sporting competition (Steca et al., 2018; Stine et al., 2019; Ionel et al., 2022). Personality traits may indirectly influence sport performance through mental skills (Fabbricatore et al., 2021). Furthermore, emotional stability and reasoning are associated with lower levels of neuroticism in successful athletes (Klatt et al., 2021). Additionally, extraversion and conscientiousness are indicated to be positively related to sport performance (Siemon and Wessels, 2022). And in general, sport experience shapes more expressive personality traits (Piepiora et al., 2022). This can be seen in the personality traits of athletes according to their sport class (Piepiora and Naczyńska, 2023). At the same time, personality traits are not a moderator of the effect of energy expenditure on physical functioning (Kekäläinen et al., 2023). Personality traits are also a certain indicator in the selection, training and psychological adjustment of athletes, where low levels of neuroticism are a determinant of performance levels (Piepiora, 2024). Therefore, it seems reasonable to verify the relationship between personality traits and athletes’ sporting level, as this area has not yet been verified.

There are three levels of sports training: amateur, competitive and professional. Amateur sport is voluntary competition undertaken for active leisure and entertainment. An amateur athlete has a hobbyist ambition to achieve life records, and this does not constitute his or her job. Competitive sport, on the other hand, is undertaken voluntarily through competition for maximum sporting performance. But competitive athletes are remunerated by working in the economic sector for their livelihood or, in the case of student athletes, are supported by scholarships. Professional sport, on the other hand, means training for sport as paid work. This can be through an employment contract with a club, a union or—in the case of athletes in the uniformed services—through their pay (Prus, 2003; Kosendiak, 2004; Pawłucki, 2015).

Human personality, temperament aside, is shaped by life experiences, interpersonal contacts, social roles, as well as strong experiences and repeated situations (Litwiniuk et al., 2019; Vaughan and Edwards, 2020). Therefore, the daily functioning of amateur, competitive and professional athletes is different. This is noticeable in the social life of athletes, and it is interesting to see how this translates into an increase in their personality traits (Afremow, 2015; Spielmann et al., 2022). Therefore, the aim of this article was to gain knowledge about the relationship between personality traits and athletes’ sporting levels. It was accepted for verification of the hypothesis that there is no relationship between personality traits and the sports level of athletes.

## Method

### Respondents

Male and female athletes (*N* = 119) took part in the experiment. Of these, 100 athletes were at a competitive level and were from Poland. Among them there were 30 basketball players, 40 football players and 30 kyokushin style karatekas. All competitive athletes were affiliated to the Polish Associations of the respective sport. The remaining 19 athletes were at the professional level. Among them were the 4-person Polish Biathlon National Team, the 7-person Polish Sledge Team, and the 8-person Swiss Mountain Bike (MTB) National Team. In professional group was elite—one Olympic medallist in biathlon and two Olympic medallists in MTB. The age of the respondents ranged between 19 and 34 years old. The quantity of the study sample is explained by its high quality.

### Research tool

The NEO-FFI Personality Questionnaire (Costa and McCrae, 2007) was used in the study. It is widely used for personality research in sport psychology. To date, most research on personality in sports has been done using this tool. Therefore, it makes sense to continue the research using the NEO-FFI, as the results obtained will be able to successfully relate to most of the studies already published. The NEO-FFI examines athletes’ personality in terms of the Big Five, i.e., five personality traits (scales): neuroticism (understood as susceptibility to experiencing negative emotions), extraversion (understood as activity in social contact with others), openness to experience (understood as curiosity about the world and openness to novelty), agreeableness (understood as attitude towards other people), conscientiousness (understood as degree of organisation, striving and perseverance) (Costa and McCrae, 2009).

The NEO-FFI is an abbreviated version of the NEO-PI-R and consists of 60 self-report statements, the truthfulness of which, in relation to oneself, is assessed by the athlete surveyed on a five-point scale. Each scale is made up of 12 questionnaire items. The results obtained allow for a complete description of the personality of the athlete under examination. The NEO-FFI is reliable and accurate. It has sten norms separately for men and women for five age groups: 15–19 years, 20–29 years, 30–39 years, 40–49 years, 50–80 years (Costa and McCrae, 2007).

The internal consistency of the NEO-FFI measurement scales used is presented in Table 1.

**Table 1:** Internal consistency of the NEO-FFI scales used.

Trait	Number of items	Cronbach’s *α*
neuroticism	12	0.89
extraversion	12	0.78
openness to experience	12	0.64
agreeableness	12	0.74
conscientiousness	12	0.87

### Procedure

The study was conducted between January and April 2024 at the Wroclaw University of Health and Sport Sciences and the Karkonosze University of Applied Sciences in Jelenia Góra based on the positive approval of the Senate Committee on Research Ethics of the Wroclaw University of Health and Sport Sciences number 20/2019. The conditions for participation in the experiment were voluntary willingness to participate in the study, possession of an athlete’s licence, documented sporting achievements and certain sporting level. Athletes willing to participate in the study, but who do not have a current athlete’s license and documented sports achievements at a given sports level, were excluded from the study. Questionnaires took place in groups of 10–15 participants for a set time of up to one hour in well-lit, noise-isolated rooms. All subjects consented to the processing of their personal data and the results obtained for the purposes of the research with full anonymity. The NEO-FFI manual was presented to the subjects before the start of the queries.

Scores on each NEO-FFI scale were calculated by summing the scores obtained by the subject for the answers, according to the key. For each answer, the subject received between 0 and 4 points, with the direction reversed for some items. As the scales contain 12 items each, the raw score on each scale ranged from 0 to 48 points. The answer keys were constructed so that a higher numerical score on a scale indicates greater severity of the trait.

### Statistical analysis

Statistical analyses were performed using IBM SPSS Statistics 29.0 software. The programme was used to analyse basic descriptive statistics, the Kruskal-Wallis’s test, post-hoc tests, Spearman’s rho pairwise correlation analyses and logistic regression. The level of statistical significance was taken as α = 0.05. Based on the data obtained, a sensitivity analysis of the statistical test was performed to find out the minimum power of the effect. With a test sample size of *N* = 119 for an α = 0.05 level and a statistical power of the test corresponding to 95% power, it is possible to detect a large effect size of η2 = 0.17 (Cohen’s *f* = 0.46).

## Results

In the first step of the analysis, basic descriptive statistics were performed together with a normality test of the distribution. Raw results were used in the analyses. It was observed that the distributions of the variables neuroticism (*p* = 0.43), openness to experience (*p* = 0.035) and conscientiousness (*p* = 0.002) deviated in shape from the normal distribution (Table 2).

**Table 2:** Basic descriptive statistics including normality of distribution test.

	*M*	*Mdn*	*SD*	*Sk.*	*Kurt.*	*Min.*	*Maks.*	*K-S*	*p*
Neuroticism	23.62	24.00	10.46	−0.20	−0.90	1.00	43.00	0.08	0.043
Extraversion	32.92	33.00	9.30	−0.40	−0.11	8.00	49.00	0.07	0.189
Openness to experience	29.44	30.00	6.82	−0.34	0.34	8.00	47.00	0.09	0.035
Agreeableness	31.89	31.00	8.22	0.05	−0.42	12.00	49.00	0.06	0.413
Conscientiousness	36.24	37.00	7.91	−0.94	0.75	9.00	50.00	0.11	0.002

*M – mean; Mdn – median; SD – standard deviation; Sk – skewness; Kurt – kurtosis; Min – the lowest value of the set; Max – the highest value of the set; K-S – Kolmogorov–Smirnov test; p – significance level.*

In the second step, the intensity of individual personality traits was compared according to sport. Due to the lack of equality in the groups, χ2(5) = 58.93, *p* < 0.001, analyses were performed using the non-parametric Kruskal-Wallis test for independent samples. The dependent variable was individual personality traits, while the independent variable was sport discipline. The analysis showed significant differences between athletes of the individual sports in the intensity of neuroticism, extraversion, openness to experience, agreeableness and conscientiousness (Table 3). This was followed by a post-hoc analysis with Bonferroni correction for multiple comparisons (Table 4).

**Table 3:** Comparison of athletes by sport in terms of individual personality traits.

	Basketball (*n* = 30)	Football (*n* = 40)	Karate (*n* = 30)	Biathlon (*n* = 4)	Luge (*n* = 7)	MTB (*n* = 8)		
Dependent variable	*Mdn*	*Mdn*	*Mdn*	*Mdn*	*Mdn*	*Mdn*	*K-W*	*p*
Neuroticism	23.50	33.50	18.50	16.50	22.00	15.00	30.10	<0.001
Extraversion	31.00	42.00	29.00	32.00	30.00	33.00	33.52	<0.001
Openness	28.00	33.00	30.00	29.00	34.00	27.50	13.57	0.019
Agreeableness	30.00	38.50	28.50	25.00	27.00	34.00	22.97	<0.001
Conscientiousness	35.00	42.00	33.50	39.50	35.00	40.00	21.40	<0.001

*Mdn – median; K-W – Kruskal-Wallis’s test; p – significance level.*

**Table 4:** Multiple comparisons of the level of intensity of individual personality traits in relation to sport.

Discipline	Neuroticism	Extraversion	Openness	Agreeableness	Conscientiousness
Karate—Luge	−0.130	−0.594	−0.848	0.447	−0.099
Karate—Basketball	1.064	1.487	−0.652	0.701	1.132
Karate—Biathlon	−0.068	0.646	−0.048	−0.908	0.983
Karate—MTB	0.792	−1.425	0.333	−1.302	−2.013
Karate—Football	−4.523^**b**^	−5.426^**b**^	−2.573	−3.849^**a**^	−4.184^**b**^
Luge—Basketball	0.524	0.321	−1.249	0.878	0.598
Luge—Biathlon	−0.145	0.151	−0.609	−0.471	0.769
Luge—MTB	−0.715	0.614	−0.944	1.364	1.468
Luge—Football	2.533	2.590	0.648	2.728^**a**^	2.365
Basketball—Biathlon	−0.584	−0.075	0.268	−1.248	0.434
Basketball—MTB	1.482	−0.460	−0.090	−0.847	−1.279
Basketball—Football	−3.386^**a**^	−3.836^**a**^	−3.270^**a**^	−3.100	−2.974^**a**^
Biathlon—MTB	0.456	−0.364	0.175	−1.635	−0.453
Biathlon—Football	−2.152	−1.843	−1.234	−2.694	−0.929
MTB—Football	−3.635^**a**^	−0.460	−1.947	−1.063	−0.541

a Significant difference between groups (*p* < 0.05) adjusted by the Bonferroni method.

b Significant difference between groups (*p* < 0.001) adjusted by the Bonferroni method.

In the next step of the analyses, the intensity of the individual personality traits was compared between athlete levels. First, basketball players, football players and karate competitors were included and combined into one sample of competitive athletes. Next, biathlonists, tobogganers and mountain bikers were included into one sample of professional athletes and the elite, i.e., Olympic Games medallists, were extracted from them. In this way, the intensity of the individual characteristics was compared between the competitive athletes (*n* = 100), the professional athletes (*n* = 19), and the elite (*n* = 3). Because the groups were not equal, χ2(2) = 139.78, *p* < 0.001, comparisons in the intensity of individual personality traits were made using the non-parametric Kruskal Wallis test for independent samples. The dependent variable was individual personality traits, while the independent variable was player level. The analysis showed that there were differences between athletes of different levels in the severity of neuroticism, H (5) = 30.10, *p* < 0.001 (Table 5). Therefore, pairwise comparisons were made along with post-hoc tests.

**Table 5:** Comparison of competitive, professional and elite athletes in terms of individual personality traits.

	Competitive athletes (*n* = 100)	Professional players (*n* = 19)	Elite players (*n* = 3)		
Dependent variable	*Mdn*	*Mdn*	*Mdn*	*K-W*	*p*
Neuroticism	25.00	22.00	8.00	9.19	0.010
Extraversion	34.50	31.00	35.00	1.72	0.422
Openness	30.50	28.00	35.00	4.42	0.110
Agreeableness	32.50	31.00	21.00	3.27	0.195
Conscientiousness	37.00	36.50	40.00	0.56	0.755

*Mdn – median; K-W – Kruskal-Wallis’s test; p – significance level.*

Post-hoc analysis with correction for multiple comparisons using the Bonferroni method (Table 6) showed statistically significant differences in neuroticism severity between the athletes (Mdn = 25.00) and the elite (Mdn =8.00).

**Table 6:** Multiple comparisons of the level of intensity of individual personality traits among athletes of different sporting levels.

Level	Neuroticism	Extraversion	Openness	Agreeableness	Conscientiousness
1–2	1.77	−0.42	−1.62	1.04	−0.620
1–3	2.57^b^	−0.91	−2.04	1.54	−0.740
2–3	1.63	1.21	1.23	0.99	0.381

b—significant difference between groups (*p* < 0.001) corrected by the Bonferroni method. Level: 1 – competitive athletes, 2 – professional athletes, 3 – elite athletes.

In the next step of the analyses, it was tested whether there was a relationship between the severity of personality traits and athlete levels. The analysis showed a weak (rho >0.3) and negative correlation between neuroticism and sporting levels rho = –0.24, *p* = 0.007 (Table 7).

**Table 7:** Correlations between personality traits and sporting level.

Variable		Level	1.	2.	3.	4.
1. Neuroticism	Spearman’s *rho*	−0.244				
	Significance	0.007				
2. Extraversion	Spearman’s *rho*	−0.08	0.242			
	Significance	0.379	0.008			
3. Openness	Spearman’s *rho*	−0.04	0.304	0.459		
	Significance	0.704	<0.001	<0.001		
4. Agreeableness	Spearman’s *rho*	−0.14	0.463	0.577	0.443	
	Significance	0.116	<0.001	<0.001	<0.001	
5. Conscientiousness	Spearman’s *rho*	−0.01	0.16	0.482	0.359	0.418
	Significance	0.945	0.075	<0.001	<0.001	<0.001

In the final step of the analyses, it was tested whether individual personality traits were related to the probability of moving to the professional level of sport. For this purpose, a logistic regression model was used, where the dependent variable was the level of athletes (1 = professional, 0 = competitive) and the independent variables were personality traits. The proposed model is statistically insignificant, meaning that there is no relationship between personality traits and the probability of turning professional, χ2(8) = 10.26, *p* = 0.247, pseudo r-square (Cox and Snell) = 0.07 (Table 8).

**Table 8:** Odds ratio (OR) for personality traits influencing the probability of transition to professionalism.

Predictor	*OR*	*CI*	*p*
Neuroticism	0.97	0.91–1.03	0.254
Extraversion	0.99	0.91–1.06	0.714
Openness	0.99	0.91–1.08	0.840
Agreeableness	0.98	0.89–1.07	0.624
Conscientiousness	1.01	0.93–1.09	0.825
Constant	1.07		0.968

*OR – odds ratio; CI – confidence interval; p – significance level.*

## Discussion

The results of the study provided cognitively interesting data. Significant differences were noted in the comparisons of footballers with other groups of athletes. Thus, football players were characterised by higher levels of neuroticism, extraversion, agreeableness and conscientiousness than karate competitors; higher levels of neuroticism, extraversion, openness to experience and conscientiousness than basketball players; higher levels of agreeableness than tobogganers; and higher levels of neuroticism than mountain bikers. The influence of the confounding variable of sample size was evident in these results. Therefore, differences were noted for the largest sample—footballers—relative to others, but not all. Namely, no significant differences were noted between footballers and biathletes, as they were similar. Therefore, the theory that the personality trait profiles shown are—in general—with lower neuroticism, higher extraversion and conscientiousness as characteristic of athletes, but at the same time are not the same as a result of the sport trained (Allen et al., 2011), has been supported. Of course, this explanation is reasonable assuming a significant effect of sport on the formation of personality traits (Lin et al., 2011; Allen et al., 2013; Trninić et al., 2016; Piepiora et al., 2022), abstracting from the levels of the sporting groups studied. Thus, it was confirmed that different sports require a slightly different intensity of personality traits specific to specific movement tasks (Steca et al., 2018; Stine et al., 2019; Ionel et al., 2022).

Verification of the personality traits of athletes from different levels was possible when the groups in question were merged into adequate samples of competitive and professional athletes, with the elite being extracted from the latter. This was reasonable in terms of identifying athletes from the levels in question. Statistically significant differences in the severity of neuroticism between the athletes and the elite were noted. This shows that top athletes—Olympic Games medallists—are distinguished by lower neuroticism intensity (Piepiora, 2024). This is also evident in the relationship noted: the lower the neuroticism, the higher the sporting level. This may indicate the attributed leading role of neuroticism severity in applied sport psychology (Balyan et al., 2016; Spielmann et al., 2024). Therefore, we concur with previous research reports on the relevance of low neuroticism intensity among athletes as a key personality trait. Lower levels of neuroticism (high levels of emotional stability) characterize athletes with great achievements (Fabbricatore et al., 2021; Klatt et al., 2021; Piepiora, 2024). But it was further noted that personality traits were not a predictor of progression to the professional level of sport. However, due to the smaller sample size of professional athletes (*n* = 19), we assume that the logistic regression model had little chance of success.

Accordingly, this experiment demonstrates the importance of neuroticism intensity, which disseminates new data and expands knowledge in sport psychology and sport theory (Otte et al., 2020). This line of research should be continued so that useful regularities can be derived for sport competition in the twenty-first century. But it should be noted that the scientific mission of researchers in this area is systematic. The problem is always the availability of top-quality athletes. Professional athletes, including elite athletes, are bound by contracts that often stipulate the dissemination of their physical and mental indicators. In addition, it is common for managers of professional athletes to demand financial fees from research institutions for the opportunity to make athletes available for scientific purposes, which also poses a problem. Therefore, the effectiveness of the scientific mission of researchers, abstracting from research funding, relates primarily to the availability of top-level athletes.

### Limitations of the research

This experiment is time-limited to 2024. Admittedly, all athletes residing in Poland at that time were given an equal opportunity to take part in the study, but only 119 athletes were able to be included within four months. Due to the relevance of the results, it was decided to disseminate them without waiting for some convenient date that might not ultimately happen. Therefore, it is recommended to continue the established line of research due to its relevance in the discipline of physical culture sciences. A strength here is the consistent use of a single tool—the NEO-FFI personality questionnaire in the Big Five model—which organises and creates a strong foundation for the adopted theoretical construct of personality in sport psychology and sport theory.

### Directions for further research

The present finding is not definitive in the direction of research on the personality of athletes in the field of sports psychology and sports theory. It is recommended to continue research queries on the largest possible populations of athletes participating in sports competition at amateur, competitive and professional levels. Only in this way is it possible to notice certain mental regularities for improving the quality and effectiveness of training and sports performance. Of particular importance here is the conduct and dissemination of research work on elite and top athletes.

### Practical applications

The present research results indicate the important role of low neuroticism among athletes. Therefore, in the mental preparation of athletes for sports competition, emphasis should be placed on developing high emotional stability. On this basis, it was assumed that during sports competition, it is important for athletes to maintain calmness in conjunction with action. Because this can translate into their sports effectiveness.

## Conclusion

There are differences between the groups of athletes studied, which may be due to different movement tasks in different sports. Different in basketball, football, kyokushin karate, biathlon, sledge, MTB. Therefore, assuming the significant influence of sports on the formation of athletes’ personalities, the distribution of the intensity of personality traits can be considered as the results of the specifics of the sport trained. But defining the study population of athletes by the sporting level of competitive, professional and elite athletes, the elite are characterised by a lower intensity of neuroticism than the competitive athletes. This translated into the following relationship: the lower the neuroticism, the higher the sporting level.

## Data Availability

The original contributions presented in the study are included in the article/supplementary material, further inquiries can be directed to the corresponding author.
